# Induced membrane technique using enriched bone grafts for treatment of posttraumatic segmental long bone defects

**DOI:** 10.1186/s10195-019-0522-6

**Published:** 2019-03-11

**Authors:** F. Piacentini, M. J. Ceglia, L. Bettini, S. Bianco, R. Buzzi, D. A. Campanacci

**Affiliations:** Department of Traumatology and General Orthopedics, Azienda Ospedaliera Universitaria Careggi, Florence, Italy

**Keywords:** Bone defect, Reconstruction, Induced membrane technique

## Abstract

**Background:**

Reconstruction of posttraumatic bone defects represents a difficult challenge. The induced membrane technique is an effective two-stage procedure for bone defect reconstruction. To overcome the problems of autologous bone grafting, different graft substitutes have been investigated. The aim of the present study is to evaluate our clinical experience in reconstruction of critical posttraumatic bone defects using an induced membrane technique based on a combination of autologous graft and allograft (cancellous bone) enriched with platelet-rich plasma (PRP) and bone marrow concentrate aspirate (BMCA).

**Materials and methods:**

Between 2009 and 2014, we reconstructed 18 posttraumatic bone defects in 16 patients. Their average length was 6.4 cm (range 1.6–13.2 cm). The defect location was the femur in nine cases (50%), the tibia in eight (44%) cases, and the humerus in one (6%) case. In all cases, we used a combination of autologous and cancellous allograft graft enriched with PRP and BMCA. Bone fixation was achieved using intramedullary nailing in 2 cases (11%), plating in 15 cases (66%), and external fixation in 1 case (6%).

**Results:**

Both clinical and radiographic union were achieved in 13 (72%) cases (13 patients). Five (28%) cases (four patients) developed nonunion. Nonunion was observed in two of eight (25%) tibial defects and in three (33%) of nine femoral defects (ns). Three of 4 (75%) double defects had delayed union, whereas 2 of 14 (14%) single defects did not heal (*p* = 0.016). The average length of the 13 defects that united was 6 cm (range 1.6–11.8 cm), while the length of the 5 defects that did not unite was 10.3 cm (range 6–13.2 cm) (*p* = 0.009).

**Conclusions:**

In this series using an induced membrane technique based on a combination of autograft and allograft enriched with BMCA and PRP, the healing rate was lower than in other series where autologous bone graft alone was employed. Nonunion was more frequent in longer and double defects. Further research aimed at developing effective alternative options to autogenous cancellous bone graft is desirable.

**Level of evidence**: III

## Introduction

A bone defect (BD) can result as a consequence of an open fracture, debridement of infection, or oncologic resection. Reconstruction of bone defects represents a challenge that can be managed using several techniques, including autologous bone grafting, distraction osteogenesis, the induced membrane technique, and vascularized bone grafts. Each technique suffers from its own difficulties and complications [1].

To date, autologous cancellous bone graft has represented the gold standard [2] for posttraumatic bone defect reconstruction, although it is limited in quantity and may result in donor-site morbidity [3, 4].

The induced membrane technique developed by Masquelet allows use of autologous grafts for management of large defects up to 25 cm [5, 6]. It is a two-stage procedure: the first stage includes debridement, positioning of a cement spacer into the bone defect, bone stabilization, and soft tissue reconstruction; the second stage includes sharp dissection of the membrane, cement removal, and autologous grafting of the defect. The induced membrane behaves as a new periosteum which preserves dead space, contains the graft, promotes vascularization, releases growth factors, and prevents graft resorption [7, 8]. Encouraging clinical results for this technique were reported by its originators [9] and confirmed by other authors [10, 11].

To overcome the problems of autologous bone grafting, namely limited graft availability and donor-site morbidity, alternative graft sources and different graft substitutes have been investigated [12, 13]. It is commonly accepted that major factors involved in bone regeneration are osteogenic cell populations, the osteoinductive stimulus, and the osteoconductive matrix [14]. Use of a scaffold enriched by stem cells and growth factors has been proposed as an alternative to autologous bone grafting [15, 16].

The aim of this study is to evaluate our clinical experience in reconstruction of critical posttraumatic bone defects using an induced membrane technique based on a combination of autologous and homologous cancellous graft enhanced with bone marrow concentrated aspirate (BMCA) and platelet-rich plasma (PRP) (Table 1).

**Table 1:** English literature case series results of long bone defect reconstruction by induced membrane technique

Author	Year	No. of defects	Average length	Graft type	Union (%)
Masquelet	2000	35	4–25 cm	Autologous	89
Obert	2015	9	5 cm (3–11 cm)	Autologous	85
Taylor	2015	69	NA	Autologous	86
Karger	2012	84	7 cm	Autologous	90
Zappaterra	2011	9	5.1 cm	Autologous (+BMP in 2 cases)	88.9
Jager	2010	39	NA	Collagen sponge + BMC (12), hydroxyapatite + BMC	92
Fernandez	2013	7	NA	Homologous + BMC	100

## Materials and methods

Between 2009 and 2014, we reconstructed 18 posttraumatic bone defects in 16 patients, including 12 (75%) males and 4 (25%) females with average age of 45 years (range 23–66 years). In all cases, the bone defect resulted from an open fracture and surgical debridement. The initial open fracture was classified as Gustilo–Anderson grade II in six cases, grade IIIA in five cases, and grade IIIB in seven cases.

Fracture was due to traffic injury in 13 (81%) cases, gunshot wound in 2 (12%) cases, and attempted suicide in 1 (6%) case.

All defects were complete. Their average length measured on postdebridement radiographs with correction for 15% magnification was 6.4 cm (range 1.6–13.2 cm). The defect location was the femur in nine cases (50%), the tibia in eight (44%) cases, and the humerus in one (6%) case. Two (12%) patients were affected by a double defect, involving the distal femur bilaterally in one case and distal femur and distal tibia in the other.

Four (22%) defects involved the diaphyseal region and 14 (78%) the metadiaphyseal region with distal involvement in 11 (61%) defects and proximal involvement in 3 (17%). Associated fractures were present in 13 (81%) patients. Fractures underwent irrigation and debridement within 8 h from trauma.

Initial fracture stabilization was achieved with external fixation in 12 (67%) cases, intramedullary nailing in 3 (17%) cases, and plating in 3 (17%) cases. The bone defect was filled with an antibiotic-loaded cement spacer. A commercially available gentamycin-loaded cement (Copal, Heraeus, Germany) was utilized. Two grams of vancomycin powder per package of cement was added. Cement overstuffing was avoided to facilitate soft tissue closure. Care was taken to place cement in and around bone stumps.

Soft tissue treatment included primary closure in 10 cases. The remaining eight cases were covered with either a vacuum-assisted closure (VAC, KCI Medical SRL, Assago, Italy) or an incision drape [17]. The decision-making criteria were excessive soft tissue tension for primary closure and availability of the VAC system. After 48 h, patients underwent a second-look procedure. Either delayed primary closure was performed (11 cases, 61%) or soft tissue reconstruction was scheduled (7 tibial fractures, 39%). The choice of the procedure was based on the dimensions of the soft tissue defect and skin viability. Soft tissue reconstruction implied rotational flap of the soleus muscle in four cases, propeller rotational flaps in two cases, and free anterolateral thigh flap in one case. The average time interval between trauma and soft tissue reconstruction was 8.5 days (range 6–12 days). The second stage of the procedure was performed after an average of 3.2 months from the injury; this interval was shorter than 2 months in 5 fractures (28%), between 2 and 4 months in 11 fractures (61%), and longer than 4 months in 2 fractures (11%). The procedure included a skin incision over the previous one, sharp dissection of the membrane, removal of cement spacer, and opening of medullary canals. Intraoperative cultural tests were drawn routinely, but only one (6%) proximal femur defect was positive for *Staphylococcus aureus*.

In all cases, we utilized a combination of autologous graft and cancellous allograft. Autologous graft was harvested from the ipsilateral anterior iliac crest. After harvesting, it was not manipulated any more. Fresh-frozen cancellous bone allograft, provided by the local bone bank, was thawed in hot saline solution and added to bulk up the defect. BMCA and PRP were employed to enrich the graft. Both were prepared according to the technique recommended by Regen Lab SA (Le Mont-sur-Lausanne, Switzerland). BMCA requires 20 cc medullary blood aspirated from the contralateral anterior iliac crest. Five cc medullary blood is drawn each time, redirecting the needle in various directions within the bone to avoid aspiration of venous blood. The blood is then inserted into dedicated tubes and centrifuged for 8 min at 3400 rpm to isolate the mesenchymal cell component.

The procedure for PRP requires 14 cc peripheral venous blood from an antebrachial vein. This drawing is centrifuged for separation of PRP. BMCA and PRP are subsequently added to the graft with addition of calcium gluconate to ensure gelification of the preparation to promote adhesion of mesenchymal cells and platelets to the graft. Ten minutes later, the graft is ready to be placed into the defect. Accurate suturing of the membrane over the graft is mandatory; usually, suction drains are not recommended.

Definitive bone fixation was achieved using intramedullary nailing in 2 cases (11%), plating in 15 cases (66%), and external fixation in 1 case (6%).

Patients were evaluated clinically and radiographically with average follow-up of 37 months (range 25–92 months). They were investigated for residual pain, joint range of motion, walking ability, deformity, and graft integration.

The radiological criterion for long bone healing was at least two healed corticals.

Statistical analysis was carried out using the chi-square and Student *t*-test, with *p* value < 0.05 considered significant.

## Results

**Fig. 1:**
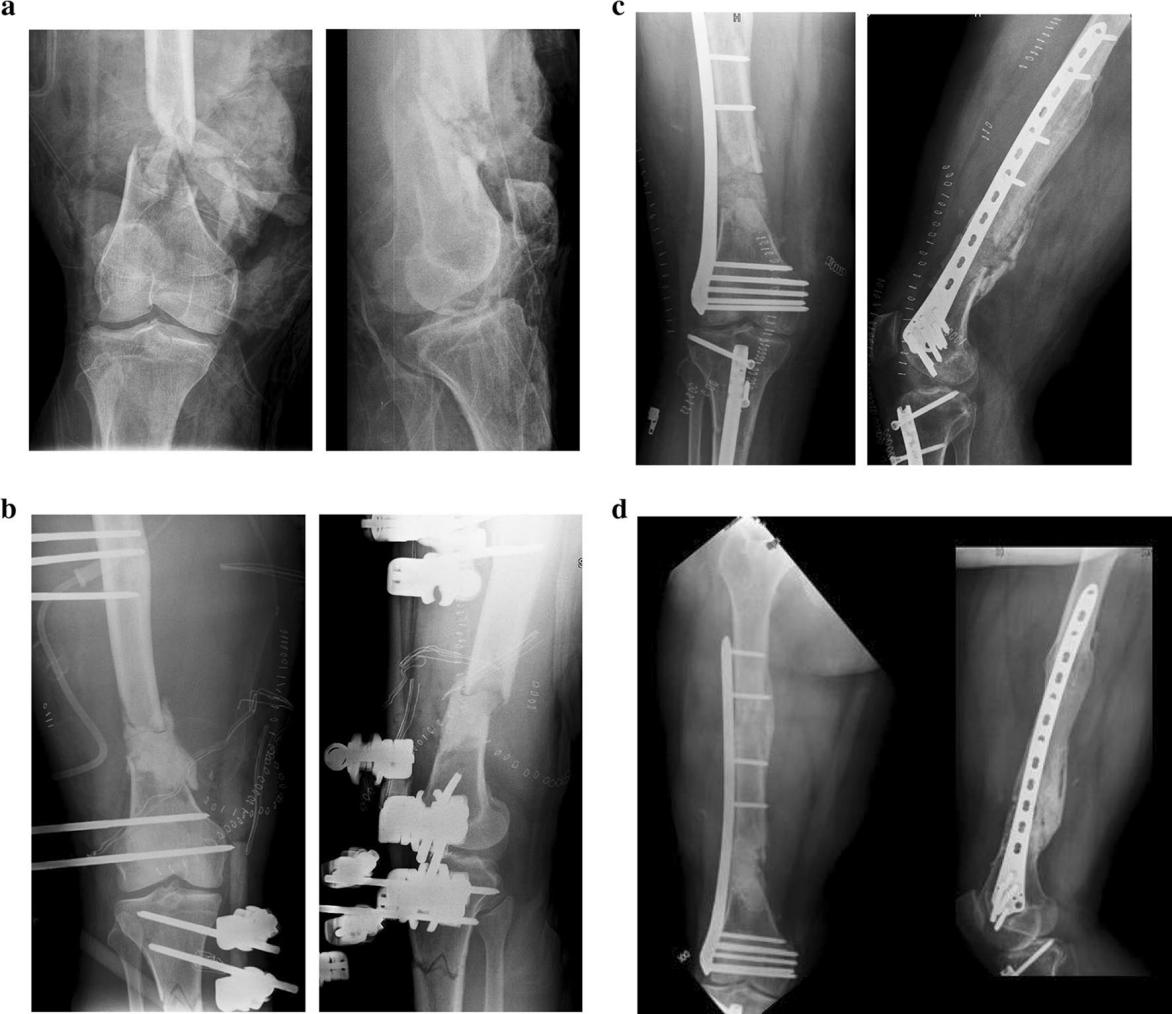
A 40-year-old man presented multifragmentary fracture of the right distal femur (**a**). He was treated with an EF and a cement spacer in the gap (**b**). After 4 months, we filled the gap with autologous graft/allograft (**c**). Follow-up at 18 months (**d**)

Both clinical and radiographic union were achieved in 13 (72%) cases in 13 patients (Fig. 1). Five (28%) cases in four patients showed delayed union. Nonunion was observed in two of eight (25%) tibial defects and in three of nine (33%) femoral defects (ns). The single humeral defect united uneventfully.

Two patients were affected by a double defect, involving the distal femur bilaterally in one case and distal femur and distal tibia in the other case. Three of 4 (75%) double defects showed delayed union, whereas 2 of 14 (14%) single defects did not heal (*p* = 0.016).

The average length of the 13 defects that united was 6 cm (range 1.6–11.8 cm), and the length of the 5 defects that did not unite was 10.3 cm (range 6–13.2 cm) (*p* = 0.009). The average age of the five patients who suffered nonunion was 51.5 years (range 28–66 years). The average age of the 11 patients bearing defects that united was 41.6 years (ns).

**Fig. 2:**
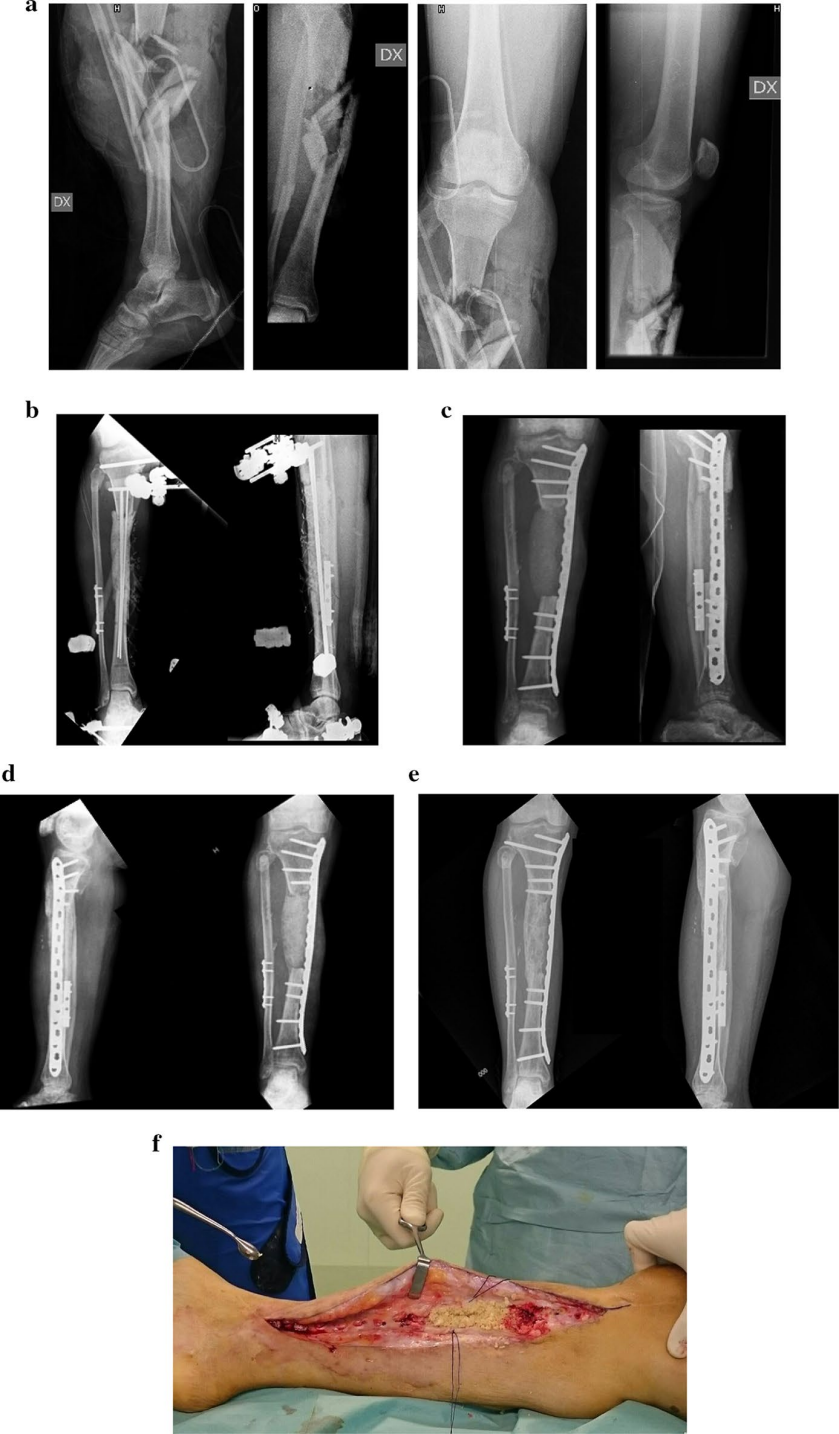
Male 49-year-old patient with grade III open fracture of the right leg (**a**). The fracture was treated with EF and placement of a cement spacer into the bone defect. Soft tissues were reconstructed at 20 days from trauma with a free anterolateral thigh flap (**b**). At 5 months, we removed the spacer, and the defect was grafted with the mixed graft and the EF was replaced by a medial locking plate (**c**). At 2 years, there were no radiological nor macroscopic signs of integration of the graft (**d**, **f**). Last follow-up (**e**)

The patients with nonunion included a 66-year-old female psychiatric patient with a grade IIIA open bilateral supracondylar femur fracture and a left patella fracture. Fixation was achieved with a plate with locking screws bilaterally. The bone defect measured 11.4 cm on the left and 6.8 cm on the right. Hardware failure was observed 7 months after implantation on the left side and 9 months on the right. The patient was treated with a distal femur resection and a cemented modular prosthesis bilaterally, with a good functional result at last follow-up. The second nonunion occurred in a 49-year-old male suffering from multiple fractures as a consequence of a traffic accident. He presented an open comminuted grade IIIA supracondylar fracture of the left femur and an open grade IIIA tibial pilon fracture with metaphyseal comminution. Fractures were initially debrided, irrigated, and temporarily stabilized with external fixator (EF). The 13.2-cm femoral defect and 4.7-cm tibial defect were filled with a cement spacer. At the second look, the EFs were removed. The distal femur was stabilized with a locking plate, while in the distal tibia fracture an intramedullary K wire was used for the fibula and an anterolateral plate for the tibia. After 3 months, the cement spacers of the femur and tibia were removed and the defects grafted. The tibial defect united uneventfully, while the femoral defect was complicated by plate failure after 8 months. The patient underwent locking plate replacement, autogenous bone grafting, and augmentation with a medial allogenic cortical strut. The bone defect eventually healed after 5 months. The third case of nonunion occurred in a 60-year-old male affected by multiple fractures including a grade IIIB open fracture of the left distal tibia which was stabilized with an EF and covered with an anterolateral thigh flap. Four months later, the bone defect was grafted and definitive fixation with an anterolateral plate undertaken. Eleven months later, radiographic evaluation demonstrated nonunion with slight varus deformity. The patient underwent autologous bone grafting and repeated fixation with consolidation of the fracture. The last case occurred in a 50-year-old man with a grade IIIB open fracture of the right tibia. The fracture was stabilized with an external fixator, and an anterolateral thigh flap was used for soft tissue reconstruction. Five months later, the 12.3-cm defect was filled with grafts and stabilized with a 4.5-mm medial plate. After 2 years, radiographic signs of incomplete healing with initial valgus deformity were evident. The plate was removed, and no macroscopic signs of osteointegration of the graft were observed (Fig. 2). The anterolateral plate was substituted, filling the defect with an autologous bone graft. At last follow-up (42 months), consolidation was achieved.

Bone union occurred without loss of reduction in 12 defects stabilized with internal fixation, while a tibial defect, stabilized with external fixation, healed with 5° deformity in the coronal plane and 10° in the sagittal.

Thirteen patients with 13 healed defects that united were also clinically evaluated. Five (38%) defects united with average shortening of 2 cm (range 1.5–3 cm). Shortening was compensated with a corresponding lift in the patient’s shoe.

Nine (69%) patients were able to ambulate independently and for as long as they desired, while three (23%) required a cane; one (7%) patient required two crutches.

The knee range of motion for patients affected by femoral defects averaged 108° (range 90–125°) with average extension lag inferior to 5°.

For patients affected by tibial defects, the average ankle range of motion was 10° in dorsiflexion (range 0–20°), 32° in plantar flexion (range 10–55°), with 50% loss of pronosupination as compared with the unaffected ankle. Five (38%) patients were free from pain, 7 (54%) complained of occasional pain, and 1 (7%) patient managed pain with daily consumption of antiinflammatory drugs.

Twelve patients (13 fractures) were satisfied with the final outcome, while four patient (5 fractures) were not satisfied.

## Discussion

We reconstructed 18 critically sized posttraumatic long bone defects using an induced membrane technique based on an autologous cancellous graft augmented with fresh-frozen bone chips, BMCA, and PRP. This combination represents a modification of the technique described by Masquelet, where autologous cancellous graft is recommended. The cited author admits augmentation with allograft chips as long as the allograft/autologous graft ratio does not exceed 1:3 [7]. In our series, the allograft/autologous graft ratio was 1:1 or higher in all cases. Graft enrichment with BMCA and PRP was supposed to increase the osteogenic and osteoinductive potential.

Bone union occurred in 13 (72%) out of 18 cases, while nonunion occurred in 5 (28%) cases. Our success rate remains inferior to the series reported by the originators of the technique, where autologous bone graft was employed [6, 18]. Such series report a union rate of 85–89%. Three out of four (75%) double defects showed delayed union, while single bone defects showed delayed union in 2 of 14 cases (14%) (*p* = 0.045). The higher nonunion rate of double defects may be explained by the greater allograft/autologous graft ratio of the bone graft. A single anterior iliac crest was used to harvest the autologous graft in single as well as double defects. Furthermore, the average length of the double defects was 9.5 cm, while it was 6 cm for the single defects (*p* < 0.0001). The larger size of the double defects and the higher allograft/autologous graft ratio may have contributed to the higher incidence of nonunion for these defects.

The nonunion rate of the femur (three out of nine cases; 33%) was greater than that for the tibia (two of eight cases; 25%), although this difference did not reach statistical significance. All three femoral nonunions occurred at the distal femur. It may be considered that reconstruction of a complete bone defect of the distal femur with a lateral locking plate may be prone to varus instability, delayed union, and fixation failure. Stability could be increased by adding a medial cortical bone strut or medial plate [19]. The average length of defects with delayed union was 10.7 cm (range 8–13.2 cm), while in those defects that united the average length was 6 cm (range 1.6–11.8 cm) (*p* = 0.009). These results suggest that larger defects suffer from a higher risk of nonunion.

We did not observe any stress fractures with this technique, as reported by other authors [6]. This may probably be due to the fixation device, which was not removed so far. For definitive osteosynthesis, we used a nail in 2 cases, a plate in 12 cases, and an external fixator in only 1 case. Stress fracture could eventually be an issue in case of hardware removal.

Management of posttraumatic bone defects using the induced membrane technique offers several advantages. Early application of a cement spacer prevents soft tissues from entering the bone defect and increases local delivery of antibiotics. It is usually possible to perform internal fixation with a plate or nails, which are more tolerable for the patient and do not interfere with soft tissue care.

An issue of concern is the amount of cancellous autograft available from each crest. It has been calculated that reconstruction of a diaphyseal tibial or femoral defect requires an average of 7 cm^3^ of autologous graft per centimeter of defect. If the defect is located in the metaphyseal region, the average amount required increases to 12 cm^3^ [20]. Anterior and posterior iliac crests provide an average of 21 and 36 cc of graft, respectively [3]. Although the posterior iliac crest provides a larger graft volume, its harvesting requires prone positioning of the patient. In this series, all operations were carried out with the patient in supine position, thus we preferred to harvest the graft from the anterior iliac crest. A diaphyseal bone defect greater than 5 cm in length requires harvesting from two or more iliac crests. The rate of complications after iliac crest harvesting varies from 0.7 to 19.4% [21].

Alternatives to iliac crest harvesting include various procedures, such as the Reamer Irrigator Aspirator (RIA) technique, bone marrow aspirate, platelet-rich plasma, allograft, and graft substitutes. The RIA technique (Synthes, Paoli, Pennsylvania) can be performed in supine position, reaming the femoral canal from the greater trochanter. It offers advantages in terms of the volume of the graft, donor-site morbidity, decreased postoperative pain, and surgical time [3]. Although RIA has a lower overall complication rate than iliac crest grafting (6% versus 19.4%), it should be highlighted that the two procedures have a similar rate of major complications (3.4% versus 4%). Major complications after use of RIA include femoral shaft fracture (1.6%) and massive bleeding [22]. A review of 27 bone defects treated by RIA graft and the Masquelet technique carried out by Stafford suggested RIA to be a valid alternative to iliac graft. On the other hand, Karger pointed out the issue of irregular ossification of the RIA graft when used alone. It is therefore advisable to use RIA graft as an augmentation of autologous graft [18].

BMCA is another option for enhancing bone healing, and can be used with autologous bone graft or with a bone graft substitute. In an experimental study [15], use of hydroxyapatite, BMCA, and PRP demonstrated the same healing potential as autologous graft. Use of hydroxyapatite and PRP or use of hydroxyapatite alone led to inferior results. In a clinical study, bone marrow concentrate was used in combination with either a collagen sponge (12 patients) or bovine hydroxyapatite (27 patients) to treat 39 bone defects [23]. At radiographic follow-up, all patients demonstrated new bone formation, but two patients underwent revision surgery due to insufficient bone healing. Complete bone healing was achieved after 17.3 weeks in the hydroxyapatite group, compared with 22.4 weeks in the collagen group. The authors concluded that BMCA combined with hydroxyapatite can reduce bone healing time when compared with BMCA with collagen sponge [16].

Platelet-rich plasma (PRP) is a technique for concentrating platelets and their growth factors to optimize bone healing. Both in vitro and in vivo experimental studies suggest the efficacy of PRP to stimulate a bone healing response, but the best approach for its clinical use remains unclear. The best method for delivery of growth factors in active form has still not been defined, and additional confounding factors include the small clinical series available and the large number of commercially available preparation kits.

Bone morphogenetic proteins are osteoinductive agents that are Food and Drug Administration (FDA) approved for spinal fusion procedures. Previous enthusiasm in literature for off-label use of BMPs in segmental bone defects has diminished more recently, mainly due to rising concerns regarding their adverse effects. In a recent study [24] carried out on 118 patients who underwent long bone nonunion surgery either with autogenous graft alone (50 patients) or with autogenous graft in combination with rhBMP-2, the authors were unable to demonstrate the desired synergistic effect of rhBMP-2.

In conclusion, reconstruction of long bone defects using the induced membrane technique appears to be a reasonable option. Autogenous cancellous bone graft still remains the gold standard. In our series, the use of a combined allograft/autogenous graft with added BMCA and PRP led to poorer results than described in literature with exclusive use of autogenous cancellous graft. Further research aimed to develop alternative options to autogenous cancellous bone graft is desirable to provide adequate amounts of graft, shorten operating times, and avoid donor-site morbidity.
